# Coronavirus Disease 2019 on the Heels of Ebola Virus Disease in West Africa

**DOI:** 10.3390/pathogens10101266

**Published:** 2021-10-01

**Authors:** Zygmunt F. Dembek, Kierstyn T. Schwartz-Watjen, Anna L. Swiatecka, Katherine M. Broadway, Steven J. Hadeed, Jerry L. Mothershead, Tesema Chekol, Akeisha N. Owens, Aiguo Wu

**Affiliations:** 1 Battelle Memorial Institute, Support to DTRA Technical Reachback, Columbus, OH 43201, USA; dembek@battelle.org (Z.F.D.); anna.l.swiatecka.ctr@mail.mil (A.L.S.); steven.j.hadeed.ctr@mail.mil (S.J.H.); tesema.chekol.ctr@mail.mil (T.C.); 2 Applied Research Associates (ARA), Support to DTRA Technical Reachback, Albuquerque, NM 87110, USA; kierstyn.t.schwartz-watjen.ctr@mail.mil (K.T.S.-W.); jmothershead@ara.com (J.L.M.); 3 Defense Sciences, Inc. (DSI), Support to DTRA Technical Reachback, San Antonio, TX 78230, USA; katherine.m.broadway.ctr@mail.mil; 4 Defense Threat Reduction Agency (DTRA), Fort Belvoir, VA 22060, USA; akeisha.n.owens.civ@mail.mil

**Keywords:** coronavirus disease 2019 (COVID-19) pandemic, Ebola outbreak, susceptible–exposed–infected–recovered (SEIR) models, vaccination, non-pharmaceutical interventions (NPIs), decision making

## Abstract

This study utilized modeling and simulation to examine the effectiveness of current and potential future COVID-19 response interventions in the West African countries of Guinea, Liberia, and Sierra Leone. A comparison between simulations can highlight which interventions could have an effect on the pandemic in these countries. An extended compartmental model was used to run simulations incorporating multiple vaccination strategies and non-pharmaceutical interventions (NPIs). In addition to the customary categories of susceptible, exposed, infected, and recovered (SEIR) compartments, this COVID-19 model incorporated early and late disease states, isolation, treatment, and death. Lessons learned from the 2014–2016 Ebola virus disease outbreak—especially the optimization of each country’s resource allocation—were incorporated in the presented models. For each country, models were calibrated to an estimated number of infections based on actual reported cases and deaths. Simulations were run to test the potential future effects of vaccination and NPIs. Multiple levels of vaccination were considered, based on announced vaccine allocation plans and notional scenarios. Increased vaccination combined with NPI mitigation strategies resulted in thousands of fewer COVID-19 infections in each country. This study demonstrates the importance of increased vaccinations. The levels of vaccination in this study would require substantial increases in vaccination supplies obtained through national purchases or international aid. While this study does not aim to develop a model that predicts the future, it can provide useful information for decision-makers in low- and middle-income nations. Such information can be used to prioritize and optimize limited available resources for targeted interventions that will have the greatest impact on COVID-19 pandemic response.

## 1. Introduction

The 2014–2016 Ebola virus disease (EVD) outbreak called global attention to existing healthcare system limitations in Guinea, Liberia, and Sierra Leone—countries where EVD raged unabated for months. Health system deficiencies included the lack of timely detection and austere response in Guinea, the epicenter of the initial outbreak. Furthermore, Guinea’s limited public health capacity as well as lack of early detection and identification of EVD may have facilitated extensive viral transmission, both nationally, and across borders to the neighboring countries of Liberia and Sierra Leone [1]. These three countries accounted for 99.9% of all Ebola outbreak cases and deaths recorded [2], and they received massive international support to combat this regional outbreak, including USD 100 million provided through the WHO’s Ebola response plan, and a USD 210 million pledge from the African Development Bank [3].

Although the West African Ebola outbreak devastated the region, the lessons learned—particularly the 2017 founding of the Africa Centers for Disease Control and Prevention (Africa CDC, Addis Ababa, Ethiopia), and increased health system funding—are positive outcomes. Coordinated efforts against the EVD outbreak contributed significantly to the development of better health policies, improvement of public health infrastructure, and increasing awareness of disease outbreaks among the wider population in this region [4]. A targeted, ring vaccination campaign was a major factor in ending the 2021 EVD outbreak in Guinea, and serves as a timely regional public health lesson for controlling coronavirus disease 2019 (COVID-19) in African nations.

The Defense Threat Reduction Agency (DTRA)’s Reachback is a multidisciplinary team that provides information and assistance to Department of Defense agencies and commands, as well as other federal agencies. Reachback was directly involved in the US government’s response to the West African EVD outbreak, providing technical and disease modeling support to US government agencies [5]. Reachback currently provides technical support for the COVID-19 pandemic response, which presents a significant public health threat to these nations—especially with the advent of a third COVID-19 pandemic wave in Africa [6,7]. 

COVID-19 outbreaks in Guinea, Liberia, and Sierra Leone are currently uncontrolled. New case reporting in these nations has increased during June and July of 2021, partly due to increased coronavirus testing capacity. Liberia experienced a surge in new COVID-19 cases in June and July of 2021, when the daily case numbers were the highest since the beginning of the pandemic. Guinea has the highest reported incidence of cases in all three countries. As of 19 July 2021, COVID-19 cases in Guinea (24,668, or 18.2 cases per 100,000) [8,9] greatly exceed those in Liberia (5396, or 10.4 cases per 100,000) [10] and Sierra Leone (6186, or 0.7 cases per 100,000) [11]. However, reported case fatality rates (CFRs) are appreciably less in Guinea (0.76%) compared to neighboring Liberia (2.74%) and Sierra Leone (1.86%). 

Steps already taken by each country to address the pandemic include total lockdowns and school closures implemented during the first waves of the COVID-19 pandemic in 2020, as well as establishing national COVID-19 task forces. Coronavirus task forces in Guinea, Liberia, and Sierra Leone follow similar contact tracing and isolation protocols to those used in China, including isolation of all individuals testing positive for COVID-19, hospitalization for the seriously ill, and sending those with mild symptoms or asymptomatic infections to specialized facilities until they test negative [12].

Coordinated international efforts have expedited COVID-19 vaccine deliveries to all three countries through the COVID-19 Vaccines Global Access (COVAX) initiative. Liberia was able to continue its vaccination campaign as a result of a shipment of 123,000 doses of AstraZeneca vaccine on 1 April 2021 [13], following an earlier shipment of 96,000 vaccine doses received on 5 March [14]. Guinea has received its first COVAX vaccines (194,400 doses of AstraZeneca vaccine), and has a projected allocation of 864,000 vaccine doses from this source [15]. Guinea also received 300,000 doses of Sinovac vaccine on 18 April 2021, and is scheduled to receive 200,000 doses of Sinopharm vaccine at a future, unannounced date [16]. Sierra Leone received 96,000 doses of AstraZeneca vaccine on 8 March 2021, out of a total allocation of 528,000 vaccine doses [17]. Sierra Leone also received 200,000 doses of Sinopharm vaccine on 25 February 2021 [18].

Given the extremely scarce resources available to combat the spread of COVID-19 in this region, our customizable modified SEIR (susceptible, exposed, infected, and recovered) model provided projections (i.e., a potential interpretation of what the future may hold for these nations). In addition to the standard compartments, this model also considered early and late disease states, isolation, treatment, and death. While not intended to forecast future events, such projections can inform decision-makers trying to allocate limited resources. This study sought to provide useful guidance for the distribution of scarce vaccines and NPI resources to control the spread of COVID-19 in these three West African nations.

## 2. Methods

The EpiGrid epidemiological model [19] used in this study has the capacity to model a variety of infectious diseases. EpiGrid is well suited to COVID-19 modeling, as it can track the geographic spread of disease in a connected regional grid without imposed administrative units (countries or regions) [19]. It can also incorporate specific infectious agent transmission details, and identify individuals throughout various states of infection or treatment, treatment modes, and different forms of NPIs [20]. The Los Alamos National Laboratories (LANL) calibrated the default COVID-19 model parameters, listed in Supplementary Table S1, using data from the early outbreaks in the city of Wuhan and eastern Hubei Province in China, as well as Italy, the United States, and Singapore [21,22,23,24,25,26,27], along with data about the healthcare response and capacity in New Mexico, USA [28]. Since the default model represents the situation in the US and similar western countries, certain parameters were adjusted to better represent the available resources in Guinea, Liberia, and Sierra Leone. The changes to the default disease model used in this study are described in the following sections.

### 2.1. Country-Specific Models

This study details the development of three separate, country-specific models reflecting the current situations in Guinea, Liberia, and Sierra Leone. To build these models, first, the default COVID-19 disease model parameters were adjusted to reflect the capacity and capabilities of the healthcare infrastructure in each country. Second, the number of infections was estimated from reported cases and deaths. Third, models were then calibrated to these estimated infections by adjusting the timing and intensity of transmission mitigations (see Section 2.2). Finally, multiple COVID-19 spread simulations of future months were conducted using various scenarios. 

Supplementary Table S2 lists all of the specific modeling parameters and assumptions for each country, but a few are described here in more detail. The estimated time of arrival for the outbreak in each country was described previously [29,30]. The number of infections required before the detection of the outbreak (threshold of detection) was set so that the date of detection in the simulations occurred on or near the day of the first reported case in each country.

**Figure 1:**
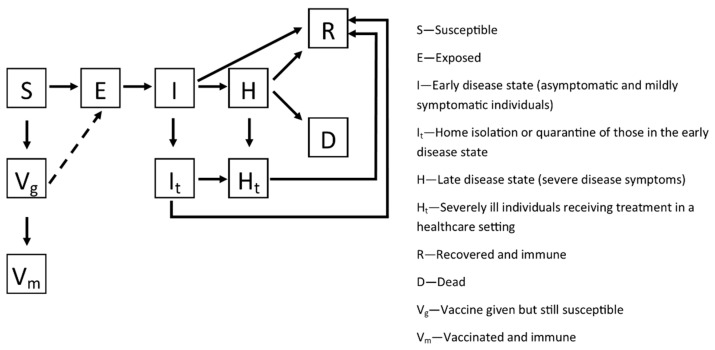
Schematic representation of the model compartments, adapted from Mourant et al. [19].

In the model, progression rates between compartments are indicated by “k” with subscripts. For example, *k_IIt_* denotes the rate of individuals moving from compartment I (early disease state) to It (home isolation/quarantine). All of the compartments are explained in Figure 1. For these three countries, little information was available about contact tracing capabilities during the current pandemic. Instead, to inform *k_IIt_*, we used data collected during the 2014 Ebola outbreak. According to a CDC study in Guinea, approximately 33% of cases had been identified as contacts [31]. Another study reported that 22.1% of confirmed cases were listed as contacts in Sierra Leone [32]. Using estimates for the number of undetected cases (see Section 2.2), the fraction of infections that would be both detected and isolated was calculated for Guinea (0.033), Liberia (0.0198), and Sierra Leone (0.01). In all three models, *k_IIt_* was increased to 0.05 after 30 days. This increase reflected the training of COVID-19 contact tracers, as reported from Sierra Leone [33].

Two parameters were scaled from the default US model, based on country-specific data: (1) the fraction of severely ill individuals that are treated, *k_HHt_*, and (2) the relative infectivity of those severely ill receiving treatment compared to those not receiving treatment. The values for *k_HHt_* in the default model were scaled according to the known numbers of beds and doctors available in each country [34,35]. Similarly, the values for relative infectivity of severely ill individuals in the default model were scaled using the Infectious Disease Vulnerability Index for each country [36].

### 2.2. Model Calibration

The EpiGrid model required the geographic location of initial infections. The number and location of initial infections differed for each model based on the availability of data for each country [37,38].

The daily number of reported cases and the total number of deaths were obtained from public sources [8,9,10,11,38]. Estimates were made of the percentage of undetected infections, which were therefore not reported in official case counts. The models used an open-source COVID-19 prevalence calculator [39] to make this estimate and then adjust the daily reported cases to account for the “true” number of daily infections. Notably, since Guinea is only reporting hospitalized deaths, the original calculator estimate was significantly different from those of the other two countries. To compensate, the reported deaths were adjusted 5-fold based on the modeling assumption that 20% of severe disease is treated. The calculator results for all three countries indicated that over 90% of infections were undetected; this is congruent with other sources [40,41].

The models did not directly incorporate individual non-pharmaceutical interventions (NPIs), due to differences in country implementation and compliance, as well as a poor understanding of how to measure their effects. Instead, by calibrating the transmission parameter beta over time, the model accounted for the effects of all NPIs and changes in behavior. Beta is the coefficient used in a pair of differential equations [18] describing the movement of individuals from susceptible (S) to exposed (E). Throughout the calibration phase of the simulations (February 2020–June 2021, Supplementary Figure S1), modifications to the beta term were added to reflect changes in NPIs. The fraction listed in Supplementary Table S2, under transmission mitigations, was multiplied by beta on the indicated day, and the modification remained in effect until another was applied. 

During calibration, two different data comparisons were made to determine when and by how much to adjust the beta term. The first comparison was between the 7-day floating average of estimated incident infections and the model results for incident infections. The second was between the estimated cumulative infections and the model results for cumulative infections. In addition to these data comparisons, current local mitigation policies were also referenced in order to determine adjustments to beta [37,42,43,44,45,46]. 

To measure calibration accuracy, a simple correlation was calculated between estimated and modeled infection datasets. The Pearson coefficients for the incident infections were 0.9472, 0.7908, and 0.8998 for Guinea, Liberia, and Sierra Leone, respectively, while the coefficients between the cumulative datasets were higher, at 0.9999, 0.9984, and 0.9982, respectively. The lower coefficients for the incident datasets were not significant (*p*-value for a two-tailed, paired *t*-test: 0.932, 0.955, and 0.813, respectively), and demonstrate the large amount of variability in daily reported cases, even with smoothing applied. The coefficients for the cumulative datasets show a significant fit between the model and the estimated cumulative infections (*p*-value for a two-tailed, paired *t*-test: 1.28 × 10^−18^, 0.050, and 2.50 × 10^−18^, respectively).

### 2.3. Vaccination

Vaccination was modeled as a single dose with 63% efficacy after 14 days. Vaccination was also concentrated geographically, with a 5–10 km radius around locations with infections, in order to approximate the vaccination strategy utilized during the 2014 Ebola outbreaks in Guinea, Liberia, and Sierra Leone [47,48]. During the calibration portion of the model, the daily rates of vaccination were set to reflect the reported number of vaccine doses administered in each country [49] (see Supplementary Table S3). Total vaccine doses administered are illustrated in Supplementary Table S4, while the percentage of the population vaccinated in each country is shown in Table 1 and Table S5. For example, the realistic scenario that we used shows the percentage of the population vaccinated (% of total population) as 8.6% in Guinea, 1.7% in Liberia, and 5.3% in Sierra Leone. Reported vaccination rates were incorporated in the calibration stage, and then dose rate assumptions were applied based on various scenarios (see Section 2.4). 

### 2.4. Simulations

After the models were calibrated, simulations of future events were run based on various scenarios to test the effects of relaxing NPIs and increasing vaccinations. Each simulation was run once in a deterministic fashion. The baseline future scenario was completed by running the calibrated model without any additional changes, and is included for purposes of comparison.

Two scenarios looking at increased rates of vaccination were included. The first, named “realistic”, shows the possible outcome if promised COVAX doses were administered by the end of the simulation. The daily vaccination rate, V, applied after the calibration phase, was calculated according to the equation:

(1) $$V=\frac{\left(Dt-Da\right)}{N}$$

where $D_{t}$ is the total doses promised by the COVAX program [13,15,17], $D_{a}$ is the total number of COVAX doses that have already been administered, and N is the number of days in the simulation after the calibration phase. The second vaccination scenario, named “optimistic”, assumed that the rate of vaccination in the realistic scenario was further amplified by the addition of 500 million doses spread across the African continent. The number of additional doses was distributed pro rata, where $P_{C}$ is the population in the country and $P_{A}$ is the population on the African continent:

(2) $$V=\left(500,000,000*\frac{P_{C}}{P_{A}}\right)+\frac{\left(Dt-Da\right)}{N}$$

The baseline vaccination scenario did not include any changes in vaccination or transmission rates after the calibration phase. In order to look at the combined effects of relaxing NPIs and increasing vaccination, the three scenarios above were repeated, but the transmission term, beta, was increased by 0.5 every 14 days until the maximum seen during the calibration phase was reached.

All simulations ran for 18 weeks past the calibration phase, or a total of 88 weeks since the date of SARS-CoV-2’s arrival in each country. In the case of Liberia, since the transmission was so high, 18 weeks captured both a rise and a fall in daily infections as the susceptible population became depleted. The lower transmission rates in both Guinea and Sierra Leone did not capture a peak within the 18-week period. Additional simulations based on the baseline scenario confirm that a slow and steady rate of transmission continues in these countries through the end of 2022, without a depletion of the susceptible population (data not shown). Longer periods were not feasible for this study, due to computational limitations.

### 2.5. Geographic Data

Hospital and clinic locations were obtained from a UN dataset [50]. The EpiGrid model provided the weekly number of cumulative infections (I) at each location on a geographic grid. The grid and population numbers were derived from the LandScan Global 2018 dataset provided by Oak Ridge National Laboratory [51].

## 3. Results

### 3.1. Model Calibration

Since many COVID-19 cases go undetected, modeling necessitates estimating the true number of infections. Based on the local public health infrastructure and the reported number of deaths, our models assumed that over 90% of infections are undetected. Models were calibrated to estimated infections through 13 June 2021 (Supplementary Figure S1). The calibrated models predicted the basic reproduction number (R_0_) for COVID-19 as 2.76, 2.1, and 2.32 for Guinea, Liberia, and Sierra Leone, respectively (see Supplementary Materials). 

Based on the modeling results, the effectiveness of NPI measures was usually visible within 1–4 weeks following implementation. In the Guinea model, the transmission rate decreased significantly during the first COVID-19 wave at the end of April 2020, 4 weeks after the lockdown began. Additionally, the transmission rate increased at the beginning of July 2020, 1 week after the reopening of schools and worship services.

The model indicates that around 17 July 2020, the number of COVID-19 cases in Liberia began to decrease. However, based on available information, the model assumed that the decrease in the reported number of cases was a result of lack of testing availability, and not due to a decrease in actual infections.

In Sierra Leone, schools closed on 31 March 2020, and the transmission rate incorporated in the model decreased one week later. Despite the introduction of inter-district travel bans and curfew on 11 April 2020, several health districts reported cases for the first time, complicating mitigation strategies. The combination of a lockdown in May 2020 and continued testing was successful at keeping disease transmission down, even after the reopening of schools on 5 October 2020.

### 3.2. Geographic Distribution

**Figure 2:**
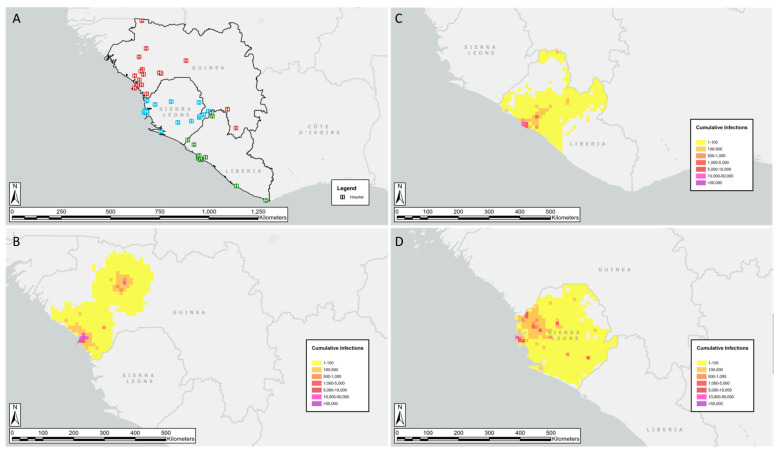
Hospital distribution across Guinea, Liberia, and Sierra Leone (**A**). Geographic distribution of cumulative infections in the countries of Guinea (**B**), Liberia (**C**), and Sierra Leone (**D**), following model calibration through 13 June 2021—approximately 70 weeks.

Models in EpiGrid have a geographic component depicting visualization of the geographic spread of an outbreak (Figure 2). In Guinea, there was almost no information about the location of cases. In contrast, the MOH situation reports from Sierra Leone provided regular case summaries by health district.

Figure 2B–D show the geographic distribution of cumulative infections near the end of the model calibration phase. Comparing the geographic spread in each country illustrates the effects of multiple SARS-CoV-2 virus-seeding (infection origin) locations. When data on case locations are available, informed decisions can be made on the geographic allocation of supplies and resources. 

### 3.3. Simulations on the Effects of Vaccination

A summary of the estimates for percentages of the total population vaccinated for each West African nation for the baseline, realistic, and optimistic vaccination scenarios are provided in Table 1. Additional summary information regarding scenario details, total vaccine doses administered, and cumulative COVID-19 infections predicted after 18 weeks in each nation can be found in the Supplementary Materials.

**Figure 3:**
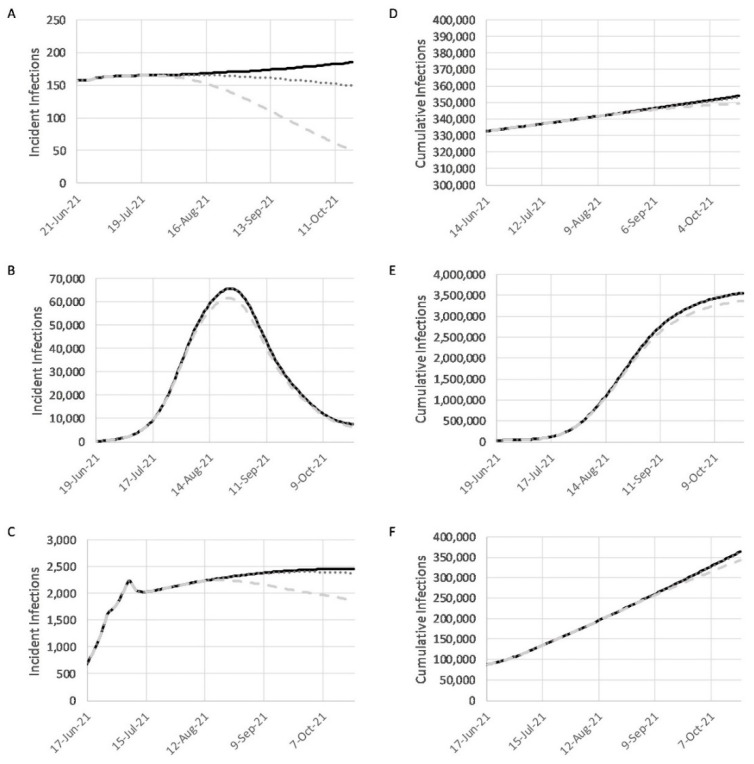
Graphs showing the projected results for vaccination scenarios for Guinea (**A**,**D**), Liberia (**B**,**E**), and Sierra Leone (**C**,**F**): (**A**–**C**) show daily incident infections; (**D**–**F**) show daily cumulative infections. The black line shows results for the baseline simulation. The dark grey, dotted line shows results for the realistic scenario when all of the promised COVAX doses are administered. The light grey, dashed line shows results for the optimistic scenario when additional vaccination is supported by national efforts or international aid.

*Baseline vaccination rate:* After calibration, the baseline vaccination simulations (solid black lines in Figure 3) were projected for 88 weeks (70-week model calibration with an 18-week projection). The vaccination rate at the end of calibration was continued unchanged for the duration of the simulation, resulting in a total of 609,640 vaccinations administered in Guinea, 230,200 vaccinations in Liberia, and 194,056 vaccinations in Sierra Leone. The baseline simulation estimated 353,960 infections on 18 October 2021 in Guinea. In Liberia, the results showed 3,553,072 infections on 23 October 2021, while in Sierra Leonne, 363,373 infections are projected on 21 October 2021. 

*Realistic vaccination rate:* This scenario examines the effects of moderately increased vaccination on controlling the spread of COVID-19. During the projection phase, vaccination rates are adjusted so that all vaccine doses promised through the COVAX program are delivered and administered by the end of 18 weeks. In Guinea, the increased rate resulted in a total 1,645,612 vaccinations. However, in Liberia, a decreased rate resulted in a total of 123,000 vaccinations. The Liberian rate decreased because Liberia has already administered many of the promised COVAX doses; thus, there were fewer to administer going forward. In Sierra Leone, the total vaccinations delivered in this scenario were 102,816.

*Optimistic Vaccination Rate:* This scenario aimed to study the effects of increasing vaccination rates even further through additional vaccine purchases by each nation, or substantial international aid. This scenario was based on the very optimistic assumption that, in addition to the COVAX program, 500 million doses of vaccine are delivered and administered across the African continent. In the model, the doses were allocated to each country based solely on population. The result in Guinea was 6,522,526 total vaccinations. In Liberia, the total was 2,000,000 vaccinations administered. In Sierra Leone, the optimistic total was 3,636,211 vaccinations administered.

Overall, the models projected fewer infections as a result of increased vaccination (Figure 3). The results for Guinea showed that the realistic vaccination scenario predicted 1087 fewer infections, while the optimistic scenario predicted 4799 fewer infections compared to the baseline total. In Sierra Leone, the trend was similar, with 1885 fewer infections in the realistic scenario, and an impressive 20,876 fewer infections in the optimistic scenario. In Liberia, the optimistic scenario produces 187,812 fewer infections. In contrast, the realistic scenario produced 4176 more infections compared to the baseline, due to decreased vaccination rates. Finally, a population-wide percentage infection reduction can also be calculated by taking the number of cumulative infections minus the number of baseline cumulative infections, and dividing by baseline cumulative infections.

### 3.4. Combined Effects of Vaccination and Relaxed NPIs under Baseline, Realistic, and Optimistic Scenarios

To study the effects of vaccination in the background of decreasing NPI compliance, we ran three additional simulations under the existing vaccination scenarios (baseline, realistic, and optimistic), but with increasing transmission rates. The scenarios used more closely reflect the reality of decreasing NPI compliance and policy changes in response to increased levels of vaccination. In these simulations, the transmission term beta was increased by 0.05 every 14 days, until the maximum transmission rate from the calibration phase was reached, or until the simulation ended (details in Supplementary Table S2).

**Figure 4:**
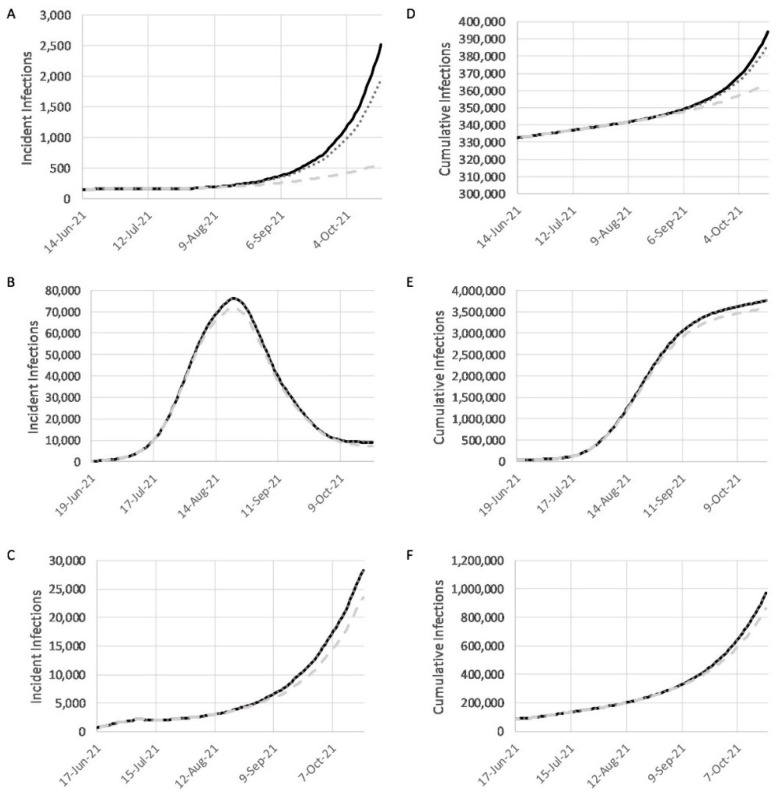
Graphs showing the results for vaccination with relaxed NPI compliance for Guinea (**A**,**D**), Liberia (**B**,**E**), and Sierra Leone (**C**,**F**): (**A**–**C**) show daily incident infections; (**D**–**F**) show daily cumulative infections. The black line shows results for the baseline simulation with relaxed NPIs. The dark grey, dotted line shows results for the realistic scenario, when all of the promised COVAX doses are administered, with relaxed NPIs. The light grey, dashed line shows results for the optimistic scenario, when additional vaccination is supported by national efforts or international aid, with relaxed NPIs.

In the baseline vaccination scenario with relaxed NPIs, there were 394,273 cumulative infections at the end of the projection period for Guinea, which was 10% greater than the cumulative infections simulated with constant NPI compliance (Figure 4). For Liberia, the result was 3,755,340 cumulative infections—a 5% increase. In Sierra Leone, the change from decreasing NPI compliance was the greatest, leading to a 63% increase that resulted in 974,184 cumulative infections.

In Guinea, comparing the vaccine scenarios with and without relaxation of NPI compliance shows an inverse trend. The realistic scenario had 8% more infections when NPIs were relaxed, while the optimistic only had 4% more infections. In Sierra Leone, the trend was similar, with 63% more infections for the realistic scenario and 61% for the optimistic scenario. In contrast, the changes for Liberia were smaller, with 5% more infections in the realistic scenario with decreasing NPI compliance, and 6% in the optimistic scenario.

## 4. Discussion

### 4.1. Vaccination

Arguably, the greatest public health tool to control a highly contagious disease outbreak such as COVID-19 is an effective vaccination campaign. Fortunately, efficacious COVID-19 vaccines are now becoming available in Africa through the COVAX initiative [52,53], Africa CDC [54], and other donation sources [55,56,57]. An important goal for COVID-19 vaccination campaigns is to rapidly vaccinate as much of the population as possible. Importantly, vaccination alone cannot halt SARS-CoV-2 transmission [58]. Since inhalation is the predominant route of transmission for COVID-19, the public is at greater risk from an infected individual. COVID-19 therefore spreads more rapidly than EVD, but is less fatal.

Initial vaccination efforts in Africa have included priority vaccination of healthcare providers and those at high risk of severe disease and death [59]. Working against government vaccination efforts in these West African nations are vaccine hesitancy and low trust of government interventions. For example, a recent survey indicated that only one in three Liberians were interested in receiving COVID-19 vaccination [60]. Given that 78% of Liberians responding to this survey indicated that they mistrust their government’s assurance that COVID-19 vaccines are safe, this vaccine skepticism is unsurprising.

Concerns over rare blood clotting that occurred in Europe from the Oxford–AstraZeneca vaccine have worked against COVAX vaccine acceptance. In response to the initial concerns over blood clotting from the Oxford–AstraZeneca vaccine, some European countries restricted vaccine distribution to certain age groups, or gave away their vaccine supply [61,62]. Following thorough investigation of this rare blood clotting disorder, the Oxford–AstraZeneca vaccine is now authorized for distribution in over 144 countries worldwide. Serious adverse events with this vaccine are notably quite rare, with 455 out of nearly 2 million vaccine doses being associated with an anaphylaxis-related adverse vaccine reaction [63]. However, information alone does not alleviate the public’s mistrust of government, particularly once concerns have become widespread on social media platforms [64].

Given global circulation of ever-emerging variant strains of SARS-CoV-2—including from Brazil, India, South Africa, and the UK—as well as the fact that many variants have the potential to spread COVID-19 more rapidly, it is imperative to vaccinate as many persons as possible against COVID-19. Current research demonstrates that the Oxford–AstraZeneca vaccine (distributed through COVAX) is partially (66%) protective against the Alpha variant (formerly: B.1.1.7 SARS-CoV-2 variant), and is also 60% effective against the Delta variant (formerly: B.1.617.2). Recent information indicates that a booster dose (i.e., vaccine dose #3) of the AstraZeneca vaccine imparts enhanced immunity against all SARS-CoV-2 variants [65].

Our models limited the role of the healthcare system to the removal of infected and contagious individuals from the general population. Such interventions primarily depend on implementing barriers to the methods of transmission, early detection and identification of infected or potentially infected individuals, and sequestration of those individuals away from the susceptible population.

As demonstrated by the modeling results, current vaccination rates reported in these nations alone are insufficient to control the spread of COVID-19. At current rates, NPIs must remain in place to protect the maximum number of individuals from becoming exposed. Our simulations showed that immunization campaigns, where scarce vaccine resources are deployed judiciously as ring vaccinations, can greatly help to contain the spread of COVID-19 in these nations. We assigned a greater daily number of vaccinations to Guinea—in part due to the greater number of vaccine doses currently allocated (>1 million), but also due to this nation’s history of successful vaccination campaigns for EVD [66].

### 4.2. Non-Pharmaceutical Interventions (NPIs)

Our model simulates a combined approximation of contact tracing, quarantine of close contacts, and school closure NPIs. It also assumes that hospitalization of infected individuals will be more effective than home isolation, since hospitalized patients are less likely to violate isolation or quarantine than those not hospitalized. However, there must be sufficient inpatient capacity, with effective infection control processes, to accept infected individuals. As demonstrated during the Ebola outbreaks and the early phase of the COVID-19 pandemic, healthcare workers are at increased risk of contracting these diseases. Since infected workers are not sequestered until diagnosed, hospitalization of all infected individuals might slow the spread of disease to the surrounding community, but is unlikely to be 100% effective.

Contact tracing and isolation of infected individuals or quarantine of close contacts should have a significant positive effect on reducing disease transmission. The CDC estimates that between 25% and 40% of infected individuals remain asymptomatic [67]. Without real time testing and immediate sequestration, a significant number of unidentified disease spreaders can remain at large. Compounding this—even among those who develop symptoms—is the long COVID-19 incubation period (up to 12 days), and disease transmission potential at least two days prior to symptoms. Finally, regardless of the COVID-19 testing method used, inaccuracies will result in a failure to identify all infected individuals. All COVID-19 tests have varying sensitivity, and can give false negatives. 

Disease transmission most often occurs through inhalation exposure to aerosols, with significantly lesser transmission due to indirect spread from contaminated surfaces [68]. Thus, physical distancing and reducing exposure to aerosols can optimize the benefits of NPIs, especially through school closures and internal movement restrictions [69].

Importantly, community participation in NPI decision making enhances NPI acceptance [70]. Implementing successful NPIs is challenging, especially for many African nations. Food scarcity, access to clean drinking water, and poverty all pose obstacles to the mitigation efforts implemented in Western countries. Complicating matters, the COVID-19 pandemic contributed to Africa’s first economic recession in 25 years [71]. Different infectious diseases can have different economic effects, unrelated to case numbers [72]. Compared to EVD, the moderately lethal but much more highly transmissible COVID-19 triggered a steep economic downturn [73]. This reflects national and international policies enacted to contain the COVID-19 pandemic [74].

Multiple NPIs that increase social distancing and strengthen healthcare systems can reduce COVID-19 case numbers and deaths. Population compliance from prior NPI adherence during the EVD outbreak may expedite many of these same measures during the pandemic. For example, school closures during 2014–2015 lasted for 5 months in Guinea [44], 6 months in Liberia [45], and 9 months in Sierra Leone [46]. Results from this study of daily incident infections and cumulative infections in Guinea, Liberia, and Sierra Leone (Figure 3 and Figure 4) demonstrate differences in the effectiveness of the various interventions. Such differences are dependent on the scale of intervention as well as the country’s preparedness level. Assessments of the effects of possible changes to mitigation strategies such as lockdowns or re-closing of schools and airports by each country resulted in thousands of fewer infections, while relaxing current mitigation efforts resulted in significant increases in the numbers of daily infections.

### 4.3. Modeling Limitations

Similar to other emerging diseases, modeling of the spread of COVID-19 is very challenging. Erroneous assumptions may become magnified when modeling countries with limited resources and restricted capabilities in their existing healthcare systems.

A major limitation is the under-reporting of COVID-19 cases, deaths, and hospitalizations. The quality of data reported by disease surveillance systems impacts the quality of modeling results. Additionally, low testing, limited laboratory capacity, and other infrastructural limitations in the modeled countries suggest that under-detection and, consequently, under-reporting of cases and deaths are significant. However, the magnitude of these problems is very difficult to quantify. Despite accounting for 17% of the world’s population, COVID-19 reporting indicates that Africa accounts for <3% of global cases and <4% of deaths. Heterogeneity in the reported data may be attributable to population demographic differences across African countries (i.e., COVID-19 mortality could be limited by the younger age structure). Despite these data limitations, early incorporation of NPIs in Africa is an important measure that potentially limited the spread of COVID-19.

Potential confounding factors for detailed analysis in the countries modeled included Guinea only reporting hospitalized COVID-19 deaths, Liberia only reporting sporadic COVID-19 deaths, and no new COVID-19 deaths reported from Sierra Leone during 29 January–8 June 2021. Therefore, COVID-19 CFR approximations in these nations are problematic. Of these three nations, COVID-19 reporting is most complete in Sierra Leone.

Demographics and incorporated measures are likely not the only factors that explain the differences between COVID-19 data reported from Africa and from other regions of the world. Poverty, food insecurity, political instability, and the public health burden from endemic diseases (e.g., malaria, tuberculosis, HIV) are existing challenges affecting the response to and impact of the COVID-19 pandemic in Africa. 

Multiple assumptions may affect disease modeling efforts, especially model calibration and estimation of the number of infections from reported cases and deaths. Based on the scarce availability of data, the incorporated COVID-19 prevalence calculator [39] provides an explicit method for estimating infections in modeling regions. However, underreporting may still constrain results.

Incomplete data on the geographical location of cases and deaths required additional assumptions for seed locations and infection distribution. In these models, the location of initial seeding was chosen based on existing reports, focused mainly on capital regions and large cities. However, it is likely that access to COVID-19 testing in the countries modeled is not evenly distributed, and may be less available in remote areas, which could lead to disproportionate numbers of cases at the regional level. Limited reporting on seroprevalence, and testing policies focused on symptomatic cases admitted to hospitals, make assessing the exact magnitude of under-detection difficult to validate. Additionally, the model design did not incorporate the effects of waning immunity after COVID-19 infection.

In the model results presented, vaccine efficacy after the first vaccine dose was estimated at 63%, and the time to build immunity was estimated at 14 days. The applied modeling methodology does not consider the effects of a second or third dose. Inability to incorporate unknown local policies regarding timelines of administering additional doses of COVID-19 vaccine is another modeling limitation.

Irregularities in vaccination rates in the modeled countries were not predicted. Our model assumed constant daily vaccination rates, continued throughout the projected timeline. However, based on available data on the progress of vaccination campaigns, significant vaccine supply shortages, insufficient numbers of healthcare workers to support the vaccination process, and day-to-day variations in vaccine delivery and administration can all occur. Our model also does not account for vaccine hesitancy consequences.

The vaccination parameters incorporated in the model were chosen based on currently available information on the types and characteristics of the COVID-19 vaccines administered. It is important to note influencing factors, such as increased vaccination manufacturing capacities and political commitment, as these dynamics may strongly impact vaccination capacity.

## 5. Conclusions

Our modeling of the effects of an improved vaccination campaign, combined with relaxation of NPIs, showed that higher vaccination rates had a greater impact on the rate of infections. A moderate vaccination rate in Guinea (realistic scenario) resulted in 2% fewer infections, while the higher rate of vaccination (optimistic scenario) reduced infections by 8% compared to the baseline scenario. For Liberia, the moderate rate of vaccination resulted in a <1% increase in infections, while the higher rate of vaccination reduced infections by 5%. In Sierra Leone, there was no effect from a moderate vaccination rate paired with NPI relaxation, but a higher vaccination rate resulted in a 10% reduction in infections over baseline.

The international support that similarly halted the Ebola epidemic—mainly via significant improvements to healthcare infrastructure and human capacity-building in these countries—could have the most impact on the spread of COVID-19. Targeted efforts should be focused on activities that demonstrate the greatest effect in reducing disease transmission. Those measures that improve the effects of vaccination—community social distancing, COVID-19 testing, and contact tracing—all appear to provide the greatest impact.

## Figures and Tables

**Table 1:** Percentage of the population vaccinated in each scenario.

	Percent of Total Population		
	Guinea	Liberia	Sierra Leone
**Baseline Vaccination**	3.5	2.2	1.7
**Realistic Vaccination**	8.6	1.7	5.3
**Optimistic Vaccination**	32	24	25

## Data Availability

Data is available upon request. Please contact the corresponding author, Aiguo Wu (aiguo.wu2.civ@mail.mil).

## Supplementary Materials

The following are available online at https://www.mdpi.com/article/10.3390/pathogens10101266/s1, Figure S1: Graphs showing the calibration phase of the simulations for Guinea (A and D), Liberia (B and E), and Sierra Leone (C and F). A–C show daily incident infections. D–F show daily cumulative infections. The black dots show the estimated infections. The grey lines show the calibrated model results, Table S1: Default COVID-19 Modeling Parameters, Table S2: Modeling parameters and assumptions employed for each country, Table S3: Summary of Vaccination Scenarios, Table S4: Total Vaccine Doses Administered, Table S5: Percent Population Vaccinated, Table S6: Cumulative COVID-19 Infections After 18-week Projection.

## References

[1] Standley C.J., Carlin E.P., Sorrell E.M., Barry A.M., Bile E., Diakite A.S., Keita M.S., Koivogui L., Mane S., Martel L.D. (2019). Assessing Health Systems in Guinea for Prevention and Control of Priority Zoonotic Diseases: A One Health Approach. One Health.

[2] 2014–2016 Ebola Outbreak in West Africa. https://www.cdc.gov/vhf/ebola/history/2014-2016-outbreak/index.html.

[3] Salaam-Blyther T. (2014). The 2014 Ebola Outbreak: International and U.S. Responses.

[4] African Countries that Faced Ebola Outbreaks Use Lessons to Fight COVID-19, Experts Say. https://www.nbcnews.com/news/world/african-countries-faced-ebola-outbreaks-use-lessons-fight-covid-19-n1181156.

[5] Dembek Z.F., Mothershead J.L., Chekol T., Myers D.B., Meris R.G., Meranus D., Wu A. (2017). Operational perspective of lessons learned from the Ebola crisis. Mil. Med..

[6] Kenya Third Wave Response: A Time to Synergize and Re-Energize. https://www.afro.who.int/news/kenya-third-wave-response-time-synergize-and-re-energize.

[7] Charts Show South Africa Coronavirus Resurgence Is Imminent. https://www.bloomberg.com/news/articles/2021-05-13/charts-show-south-africa-coronavirus-resurgence-is-imminent.

[8] Guinea, World Health Organization. https://covid19.who.int/region/afro/country/gn.

[9] Worldometer, Guinea. https://www.worldometers.info/coronavirus/country/guinea/.

[10] Liberia, World Health Organization. https://covid19.who.int/region/afro/country/lr.

[11] World Health Organization Sierra Leone. https://covid19.who.int/region/afro/country/sl.

[12] Maxmen A. (2020). Ebola prepared these countries for coronavirus–but now they are floundering. Nature.

[13] Ministry of Health Starts Vaccination Campaign Against COVID-19. https://www.unicef.org/liberia/press-releases/ministry-health-starts-vaccination-campaign-against-covid19-0.

[14] Liberia: COVID-19 Vaccine Is Finally Here. https://frontpageafricaonline.com/editorial/liberia-covid-19-vaccine-is-finally-here/.

[15] COVAX Vaccine Roll-Out–Guinea. https://www.gavi.org/covax-vaccine-roll-out/guinea.

[16] Guinea Receives Purchase of 300,000 Sinovac COVID-19 Vaccines. https://www.reuters.com/business/healthcare-pharmaceuticals/guinea-receives-purchase-300000-sinovac-covid-19-vaccines-2021-04-18/.

[17] COVAX Vaccine Roll-Out–Sierra Leone. https://www.gavi.org/covax-vaccine-roll-out/sierra-leone.

[18] China Donates 200,000 Doses of COVID-19 Vaccine to Sierra Leone. http://www.xinhuanet.com/english/2021-02/26/c_139767646.htm.

[19] Mourant J.R., Fenimore P.W., Manore C.A., McMahon B.H. (2018). Decision Support for Mitigation of Livestock Disease: Rinderpest as a Case Study. Front. Vet. Sci..

[20] Tyler C. What Happens Next, Los Alamos National Laboratory. https://www.lanl.gov/discover/publications/1663/2021-february/what-happens-next.shtml.

[21] Guan W.J., Ni Z.Y., Hu Y., Liang W.H., Ou C.Q., He J.X., Liu L., Shan H., Lei C.L., Hui D.S.C. (2020). Clinical Characteristics of Coronavirus Disease 2019 in China. N. Engl. J. Med..

[22] Wang D., Hu B., Hu C., Zhu F., Liu X., Zhang J., Wang B., Xiang H., Cheng Z., Xiong Y. (2020). Clinical Characteristics of 138 Hospitalized Patients With 2019 Novel Coronavirus-Infected Pneumonia in Wuhan, China. JAMA.

[23] Lavezzo E., Franchin E., Ciavarella C., Cuomo-Dannenburg G., Barzon L., Del Vecchio C., Rossi L., Manganelli R., Loregian A., Navarin N. (2020). Suppression of a SARS-CoV-2 outbreak in the Italian municipality of Vo’. Nature.

[24] Li Q., Guan X., Wu P., Wang X., Zhou L., Tong Y., Ren R., Leung K.S.M., Lau E.H.Y., Wong J.Y. (2020). Early Transmission Dynamics in Wuhan, China, of Novel Coronavirus-Infected Pneumonia. N. Engl. J. Med..

[25] Arons M.M., Hatfield K.M., Reddy S.C., Kimball A., James A., Jacobs J.R., Taylor J., Spicer K., Bardossy A.C., Oakley L.P. (2020). Presymptomatic SARS-CoV-2 Infections and Transmission in a Skilled Nursing Facility. NEJM.

[26] Bullard J., Dust K., Funk D., Strong J.E., Alexander D., Garnett L., Boodman C., Bello A., Hedley A., Schffman Z. (2020). Predicting Infectious Severe Acute Respiratory Syndrome Coronavirus 2 from Diagnostic Samples. Clin. Infect. Dis..

[27] van Kampen J.J.A., van de Vijver D.A.M.C., Fraaij P.L.A., Haagmans B.L., Lamers M.M., Okba N., van den Akker J.P.C., Endeman H., Gommers D.A.M.P.J., Cornelissen J.J. (2021). Duration and Key Determinants of Infectious Virus Shedding in Hospitalized Patients with Coronavirus Disease-2019 (COVID- 19). Nat. Commun..

[28] New Mexico Department of Health, COVID-19 in New Mexico. https://cvprovider.nmhealth.org/public-dashboard.html.

[29] Adiga A., Venkatramanan S., Schlitt J., Peddireddy A., Dickerman A., Bura A., Warren A., Klahn B.D., Mao C., Xie D. (2020). Evaluating the Impact of International Airline Suspensions on the Early Global Spread of COVID-19. medRxiv.

[30] Brockmann D., Helbing D. (2013). The hidden geometry of complex, network-driven contagion phenomena. Science.

[31] Dixon M.G., Taylor M.M., Dee J., Hakim A., Cantey P., Lim T., Bah H., Camara S.M., Ndongmo C.B., Togba M. (2015). Contact Tracing Activities during the Ebola Virus Disease Epidemic in Kindia and Faranah, Guinea, 2014. Emerg. Infect. Dis..

[32] Olu O.O., Lamunu M., Nanyunja M., Dafae F., Samba T., Sempiira N., Kuti-George F., Abebe F.Z., Sensasi B., Chimbaru A. (2016). Contact Tracing during an Outbreak of Ebola Virus Disease in the Western Area Districts of Sierra Leone: Lessons for Future Ebola Outbreak Response. Front. Public Health.

[33] Sierra Leone Ebola Lessons Shaping Massachusetts COVID-19 Response. https://www.pih.org/article/sierra-leone-ebola-lessons-shaping-massachusetts-covid-19-response.

[34] The World Factbook. https://www.cia.gov/the-world-factbook/.

[35] Hospital Beds (per 1,000 People). https://data.worldbank.org/indicator/SH.MED.BEDS.ZS.

[36] Moore M., Gelfeld B., Okunogbe A.T., Paul C. (2016). Identifying Future Disease Hot Spots: Infectious Disease Vulnerability Index.

[37] Republic of Sierra Leone, Ministry of Health Website. https://mohs.gov.sl/covid-19/..

[38] Skrip L.A., Selvaraj P., Hagedorn B., Ouédraogo A.L., Noori N., Orcutt A., Mistry D., Bedson J., Hébert-Dufresne L., Scarpino S.V. (2021). Seeding COVID-19 across Sub-Saharan Africa: An Analysis of Reported Importation Events across 49 Countries. Am. J. Trop. Med. Hyg..

[39] COVID-19 Prevalence Calculator. https://preventepidemics.org/covid19/resources/prevalence-calculator/#how-to-use-the-calculator.

[40] Update on COVID-19 in Africa. https://preventepidemics.org/covid19/science/insights/update-on-covid-19-in-africa/.

[41] IHME, COVID-19 Results Briefing: Global. http://www.healthdata.org/sites/default/files/files/Projects/COVID/2021/briefing_Global_20210122.pdf/.

[42] Partnership for Evidence-Based Response to COVID-19 (PERC)-Effective Implementation of Public Health and Social Measures in Liberia: Situational Analysis Data, 30 April 2020. https://preventepidemics.org/wp-content/uploads/2020/05/Liberia_perc-countrybrief_mobility.pdf.

[43] Cuccaro F., Debenedetti L., Holzinger A. New RECOVR Survey Findings in Sierra Leone Highlight Socio-Economic Fallout from COVID-19 as New Restrictions Are Put in Place. https://www.poverty-action.org/blog/new-recovr-survey-socio-economic-fallout.

[44] Ebola Crisis: Guinea Schools Reopen after Five-Month Closure. https://www.bbc.com/news/world-africa-30879937.

[45] Rodriguez M. 5 Tips for Reopening after COVID-19 School Closures from a Liberian School that Made It through Ebola. https://www.nwea.org/blog/2020/5-tips-reopening-after-covid-19-from-liberian-school-made-through-ebola/.

[46] Fofana U. Sierra Leone Schools Reopen after Long Closure Due to Ebola. https://www.reuters.com/article/us-health-ebola-leone-education/sierra-leone-schools-reopen-after-long-closure-due-to-ebola-idUSKBN0N51JY20150414.

[47] ‘Make Ebola a Thing of the Past’: First Vaccine against Deadly Virus Approved. https://www.nature.com/articles/d41586-019-03490-8.

[48] Ebola Vaccination Starts in Guinea to Curb New Outbreak. https://www.afro.who.int/news/ebola-vaccination-starts-guinea-curb-new-outbreak.

[49] Coronavirus (COVID-19) Vaccinations-Statistics and Research. https://ourworldindata.org/covid-vaccinations.

[50] United Nations Office for the Coordination of Humanitarian Affairs: HDX Human Data Set. https://data.humdata.org/dataset/.

[51] LandScan Datasets. https://landscan.ornl.gov/landscan-datasets.

[52] COVAX, Working for a Global Equitable Access to COVID-19 Vaccines. https://www.who.int/initiatives/act-accelerator/covax.

[53] Berkley S. COVAX Explained. https://www.gavi.org/vaccineswork/covax-explained.

[54] Africa CDC, African Union. https://africacdc.org/.

[55] Guinea Gets Donation of 200,000 Doses of COVID-19 Vaccine from China. https://www.reuters.com/article/health-coronavirus-guinea/guinea-gets-donation-of-200000-covid-19-vaccine-doses-from-china-idUSL5N2L1637.

[56] Liberia: MTN Group Donates 27,000 COVID-19 Vaccines to Government. https://frontpageafricaonline.com/news/liberia-mtn-group-donates-27000-covid-19-vaccines-to-government/.

[57] Sierra Leone Thanks China for Vaccine Donation. https://news.cgtn.com/news/2021-03-02/Sierra-Leone-thanks-China-for-vaccine-donation-YjfdPpiABq/index.html.

[58] Roberts M., BBC Visual Journalism Team Vaccines Alone Will Not Stop Covid Spreading–Here’s Why. https://www.bbc.co.uk/news/resources/idt-40ac92b1-1750-4e86-9936-2cda6b0acb3f.

[59] Nachega J.B., Sam-Agudu N.A., Masekela R., van der Zalm M.M., Nsanzimana S., Condo J., Ntoumi F., Rabie H., Kruger M., Wiysonge C.S. (2021). Addressing challenges to rolling out COVID-19 vaccines in African countries. Lancet Glob. Health.

[60] Seydou A. AD432: Who Wants COVID-19 Vaccination? In 5 West African Countries, Vaccine Hesitancy Is High, Trust Is Low. https://afrobarometer.org/publications/ad432-who-wants-covid-19-vaccination-5-west-african-countries-hesitancy-high-trust-low.

[61] AstraZeneca Vaccine: Denmark Stops Rollout Completely. https://www.bbc.com/news/world-europe-56744474.

[62] Mueller B. AstraZeneca Vaccine Faces New Setbacks in UK and European Union. https://www.nytimes.com/2021/04/07/world/europe/astrazeneca-uk-european-union.html.

[63] Jeong M. Oxford-AstraZeneca Vaccine: What to Know about Side Effects. https://www.medicalnewstoday.com/articles/oxford-astrazeneca-vaccine-what-to-know-about-side-effects.

[64] Samarasekera U. (2021). Feelings toward COVID-19 vaccination in Africa. Lancet Infect. Dis..

[65] DeFeudis N. COVID-19 Roundup: Study Suggests AstraZeneca/Oxford Booster Is Effective against Variants–Report; IFPMA Issues Guidance on Vaccine Inequity. https://endpts.com/covid-19-roundup-study-suggests-astrazeneca-oxford-booster-is-effective-against-variants-report-ifpma-issues-guidance-on-vaccine-inequity/.

[66] Weyer J. A Recent Ebola Outbreak Puts Guinea’s “Muscle Memory” to the Test. https://qz.com/africa/1977293/why-has-ebola-returned/.

[67] Pandemic Planning Scenarios. https://www.cdc.gov/coronavirus/2019-ncov/hcp/planning-scenarios.html.

[68] Coronavirus 19: How COVID-19 Spreads. https://www.cdc.gov/coronavirus/2019-ncov/prevent-getting-sick/how-covid-spreads.html.

[69] Jefferson T., Del Mar C.B., Dooley L., Ferroni E., Al-Ansary L.A., Bawazeer G.A., van Driel M.L., Jones M.A., Thorning S., Beller E.M. (2020). Physical interventions to interrupt or reduce the spread of respiratory viruses. Cochrane Database Syst. Rev..

[70] Seale H., Dyer C.E.F., Abdi I., Rahman K.M., Sun Y., Qureshi M.O., Dowell-Day A., Sward J., Islam M.S. (2020). Improving the impact of non-pharmaceutical interventions during COVID-19: Examining the factors that influence engagement and the impact of individuals. BMC Infect. Dis..

[71] Africa Faces First Recession in 25 Years Amid COVID-19 Pandemic: UN Report. http://www.xinhuanet.com/english/africa/2021-03/02/c_139778665.htm.

[72] Stoop N., Desbureaux S., Kaota A., Verpoorten M. (2021). COVID-19 vs. Ebola: Impact on households and small businesses in North Kivu, Democratic Republic of Congo. World Dev..

[73] Egger D., Miguel E., Warren S.S., Shenoy A., Collins E., Karlan D., Parkerson D., Mobarak A.M., Fink G., Udry C. (2021). Falling living standards during the COVID-19 crisis: Quantitative evidence from nine developing countries. Sci. Adv..

[74] Aktar M.A., Alam M.M., Al-Amin A.Q. (2021). Global economic crisis, energy use, CO_2_ emissions, and policy roadmap amid COVID-19. Sustain. Prod. Consum..

