# Supplementary material for: Coronavirus Disease 2019 on the Heels of Ebola Virus Disease in West Africa
Source: Pathogens. 2021 Oct 1;10(10):1266. doi: 10.3390/pathogens10101266 (PMC8537256; doi:10.3390/pathogens10101266)
Supplement: Supplementary file 1 [file pathogens-10-01266-s001.zip › pathogens-1344302-supplementary.pdf]

Supplemental Methods

The default disease parameters, developed by Los Alamos National Labs, for the EpiGrid [1], COVID-19 model were based upon early outbreaks in many countries. Reports from Wuhan indicate the mean time from exposure to symptom onset is 4 days [2,3]. Early data from New Mexico and a study from Italy identified that about 20% of cases develop severe symptoms that warrant hospitalizations [4,5]. Reports from Wuhan also indicate that the mean time between becoming infectious and being hospitalized was 8 days [3,6]. Viable virus was found in a variety of specimens 8-20 days after symptom onset [7–9]. Based on data from Wuhan the median hospital stay was 10 days while data from the US indicates longer hospital stays.

Supplemental Table S1. Default COVID-19 Modeling Parameters.

Parameter	Default Value ($days^{-1}$) and Justification	Reference
k_{EI} ($days^{-1}$)	1/4 days = 0.25 $days^{-1}$	[2, 3]
k_{IH} ($days^{-1}$)	0.2/8 days = 0.025 $days^{-1}$	[3, 4, 6]
k_{IR} ($days^{-1}$)	0.8/8 days = 0.1 $days^{-1}$	[3, 4, 6]
k_{ItH} ($days^{-1}$)	0.2/7 days = 0.0285714 $days^{-1}$	[3, 4, 6]
k_{ItR} ($days^{-1}$)	0.8/7 days = 0.1142857 $days^{-1}$	[3, 4, 6]
k_{HD} ($days^{-1}$)	0.5/4 days = 0.125 $days^{-1}$	[7, 8, 9]
k_{HR} ($days^{-1}$)	0.5/4 days = 0.125 $days^{-1}$	[7, 8, 9]
k_{HtD} ($days^{-1}$)	0.2/13.2 days = 0.0152 $days^{-1}$	[5, 7, 8, 9]
k_{HtR} ($days^{-1}$)	0.8/13.2 days = 0.0606 $days^{-1}$	[5, 7, 8, 9]
Relative Infectivity of H	0.01: Severely ill individuals receiving treatment are 100 times less likely to infect others	*
Fraction of H Treated (k_{HHt})	98 of every 100 severely ill individuals receive treatment	*
Fraction of I in Isolation/Quarantine (k_{IIt})	No individuals are identified and isolated prior to onset of severe illness	**

* Assumptions based on model calibrations, data not presented

** Assumption reflects the situation early in an outbreak

Supplemental Table S2. Modeling parameters and assumptions employed for each country.

Parameter	Guinea	Liberia	Sierra Leone	Reference
Infections Reported (percent)	7	6	5	[10]

Prevalence Calculator	IFR: 0.24, mid-point estimate*	IFR: 0.25, mid-point estimate	IFR: 0.28, high estimate	[10]
Initial Location	Conakry Airport	Monrovia	Freetown, Western Area Urban District	[11, 12]
Estimated Arrival Time	10 February 2020	15 February 2020	13 February 2020	[13, 14]
Day of Detection	14 March 2020 (day 33)	16 March 2020 (day 30)	31 March 2020 (day 47)	Calculated result
Later Seeding Locations (day of seeding)	Labé (40)	Kakata (1), Sanniquellie (30), Voinjana (30), Gbarnga (75)	15 health districts: Port Loko (7), Western Area Rural (14), Kenema (32), Tokolili (36), Bombali (53), Bonthe (53), Bo (53), Koinnadugu (58), Falaba (58), Kailahun (60), Moyamba (62), Pujehun (65), Kono (69), Kambia (71), Karene (78)	[11, 12]
Beta	0.330	0.250	0.253	Based on calibration
Transmission Mitigations (days after detection, fractions override those listed on previous days and apply to the entire country)	0.50 on day 40<br>0.40 on day 49<br>0.35 on day 70<br>0.40 on day 109<br>0.35 on day 154<br>0.50 on day 210<br>0.40 on day 223<br>0.35 on day 231<br>0.30 on day 237<br>0.49 on day 288	1.3 on day 35<br>0.49 on day 46<br>0.87 on day 85<br>0.43 on day 116<br>0.28 on day 146<br>0.26 on day 182<br>0.54 on day 201<br>0.57 on day 235<br>0.79 on day 253<br>0.34 on day 289	0.70 on day 15<br>0.60 on day 50<br>0.50 on day 60<br>0.23 on day 70<br>0.35 on day 87<br>0.65 on day 160<br>0.30 on day 190<br>0.9825 on day 255<br>0.10 on day 305<br>0.30 on day 340	Based on calibration

	0.62 on day 335<br>0.75 on day 338<br>0.60 on day 355<br>0.50 on day 359<br>0.38 on day 370<br>0.35 on day 380<br>0.30 on day 384<br>0.27 on day 403<br>0.40 on day 460<br>0.425 on day 470	0.54 on day 375<br>0.45 on day 415<br>0.75 on day 425<br>0.85 on day 432<br>1.5 on day 477	0.52 on day 395<br>0.75 on day 405<br>0.10 on day 430	
R_0	2.76	2.1	2.32	Model result
k_{HHt}**	0.2	0.2	0.135	[15, 16]
k_{IIt}**[#]	0.033	0.0198	0.0100	[17, 18, 19]
Relative Infectivity of H**	0.0434	0.0400	0.0414	[20]
Vaccination start date	15 March 2021	01 April 2021	22 March 2021	[21]
Vaccination Rates (doses per day, days after model start)	3,500 on day 399<br>1,650 on day 416<br>850 on day 430<br>5,000 on day 450<br>1,500 on day 462<br>9,250 on day 467<br>2,420 on day 479	800 on day 411<br>1,400 on day 454<br>400 on day 463<br>1,000 on day 466<br>100 on day 468	3,558 of day 403<br>3,033 on day 406<br>6,053 on day 411<br>579 on day 412<br>1,185 on day 422<br>1,928 on day 426<br>1,302 on day 428<br>976 on day 437<br>235 on day 439<br>661 on day 441<br>643 on day 450	Calculated from [21]

			464 on day 473<br>2,525 on day 481<br>816 on day 483	

* Death input for calculator was increased 5-fold

**Implemented on the day of detection according to each model

Adjusted to 0.05 30 days after detection

Calculated result from the model

Supplemental Results

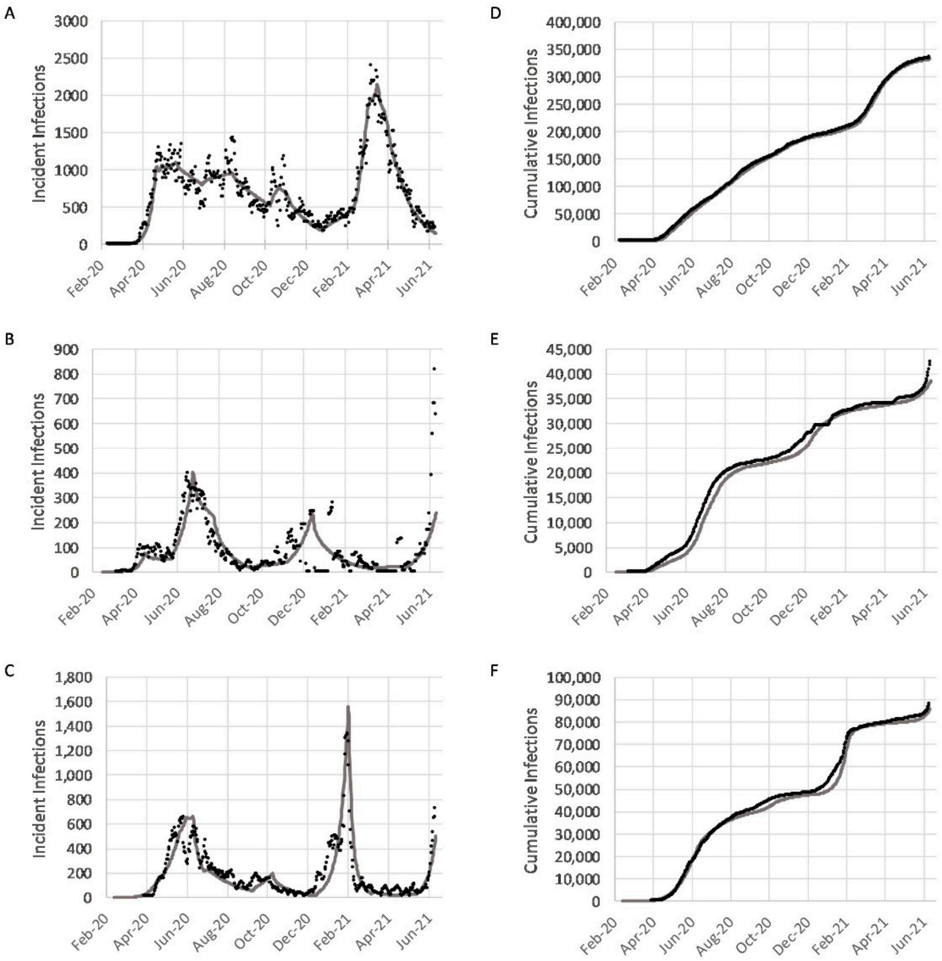

Supplemental Figure S1. Graphs showing the calibration phase of the simulations for Guinea (A and D), Liberia (B and E), and Sierra Leone (C and F). A-C show daily incident infections. D-F show daily cumulative infections. The black dots show the estimated infections. The grey lines show the calibrated model results.

Simulations on the effects of vaccination

To study the effects of vaccination, especially when supported by national purchases or global aid, we ran three additional simulations representing different vaccination rates described in Table 3.

Supplemental Table S3. Summary of Vaccination Scenarios.

Parameter	Guinea	Liberia	Sierra Leone
Scenarios Begin (day 490)	June 14	June 19	June 17
Projection Ends (week 88, day 616)	October 18	October 23	October 21
Maximum Transmission Mitigation During Projection	0.75 on day 588	1.95 on day 616	1.075 on day 616
Baseline Vaccination Rate	2,420 doses per day	800 doses per day	820 doses per day
Realistic Vaccination Rate (COVAX)	10,642 doses per day	470 doses per day	5,049 doses per day
Optimistic Vaccination Rate (COVAX+ Additional Aid)	49,348 doses per day	15,450 doses per day	28,858 doses per day

Vaccination Data

Supplemental Table S4. Total Vaccine Doses Administered

	Guinea	Liberia	Sierra Leone
**Baseline**	446,827	110,472	133,096
**Realistic Vaccination**	1,095,850	86,035	404,676
**Optimistic Vaccination**	4,151,393	1,214,919	1,928,719
**Baseline + Relaxed NPI**	446,636	107,701	133,036
**Realistic Vaccination + Relaxed NPI**	1,095,143	84,384	404,248
**Optimistic Vaccination + Relaxed NPI**	4,149,839	1,161,442	1,925,756

Supplemental Table S5. Percent Population Vaccinated.

	Guinea	Liberia	Sierra Leone
**Population**	12,771,246	4,937,374	7,650,150
**Baseline (percent)**	3.50	2.24	1.74
**Realistic Vaccination (percent)**	8.58	1.74	5.29
**Optimistic Vaccination (percent)**	32.51	24.61	25.21

**Baseline + Relaxed NPI (percent)**	3.50	2.18	1.74
**Realistic Vaccination + Relaxed NPI (percent)**	8.58	1.71	5.30
**Optimistic Vaccination + Relaxed NPI (percent)**	32.49	23.52	25.17

Supplemental Table S6. Cumulative COVID-19 Infections After 18-week Projection

	**Guinea**	**Liberia**	**Sierra Leone**
**Baseline**	353,960	3,553,072	363,373
**Realistic Vaccination**	353,873	3,557,248	361,488
**Optimistic Vaccination**	349,161	3,365,260	342,497
**Baseline + Relaxed NPI**	394,273	3,755,340	974,184
**Realistic Vaccination + Relaxed NPI**	386,633	3,759,340	974,184
**Optimistic Vaccination + Relaxed NPI**	364,085	3,576,702	867,116

SUPPLEMENTAL REFERENCES

1. Mourant, J.R.; Fenimore, P.W.; Manore, C.A.; McMahon, B.H.; Decision Support for Mitigation of Livestock Disease: Rinderpest as a Case Study. *Front Vet Sci.* **2018**, *5:182*. DOI: 10.3389/fvets.2018.00182.
2. Guan, W.J.; Ni, Z.Y.; Hu, Y.; Liang, W.H.; Ou, C.Q.; He, J.X.; Liu, L.; Shan, H.; Lei, C.L.; Hui, D.S.C.; Du, B.; Li, L.J.; Zeng, G.; Yuen, K.Y.; Chen, R.C.; Tang, C.L.; Wang, T.; Chen, P.Y.; Xiang, J. Li, S.Y.; Wang, J.L.; Liang, Z.J.; Peng, Y.X.; Wei, L.; Liu, Y.; Hu, Y.H.; Peng, P.; Wang, J.M.; Liu, J.Y.; Chen, Z.; Li, G.; Zheng, Z.J.; Qiu, S.Q.; Luo, J.; Ye, C.J.; Zhu, S.Y.; Zhong, N.S.; China Medical Treatment Expert Group for Covid-19. Clinical Characteristics of Coronavirus Disease 2019 in China. *N Engl J Med.* **2020,** 382:18, 1708-1720. DOI: 10.1056/NEJMoa2002032.
3. Wang, D.; Hu, B.; Hu, C.; Zhu, F.; Liu, X.; Zhang, J.; Wang, B.; Xiang, H.; Cheng, Z.; Xiong, Y.; Zhao, Y.; Li, Y.; Wang, X.; Peng, Z. Clinical Characteristics of 138 Hospitalized Patients With 2019 Novel Coronavirus-Infected Pneumonia in Wuhan, China. *JAMA*. **2020**, *323:11,* 1061-1069. DOI: 10.1001/jama.2020.1585. Erratum in: *JAMA*. **2021**, *325:11*, 1113.
4. Lavezzo, E.; Franchin, E.; Ciavarella, C.; Cuomo-Dannenburg, G.; Barzon, L.; Del Vecchio, C.; Rossi, L.; Manganelli, R.; Loregian, A.; Navarin, N.; Abate, D.; Sciro, M.; Merigliano, S.; De Canale, E.; Vanuzzo, M.C.; Besutti, V.; Saluzzo, F.; Onelia, F.; Pacenti, M.; Parisi, S.G.; Carretta, G.; Donato, D.; Flor, L.; Cocchio, S.; Masi, G.; Sperduti, A.; Cattarino, L.; Salvador, R.; Nicoletti, M.; Caldart, F.; Castelli, G.; Nieddu, F.; Labella, B.; Fava, L.; Drigo, M, Gaythorpe, K.A.M.; I.C.C.-. R. Team; Brazzale, A.R.; Toppo, S.; Trevisan, M.; Baldo, V.; Donnelly, C.A.; Ferguson, N.M.; Dorigatti, I.; Crisanti, A. Suppression of a sars-cov-2 outbreak in the Italian municipality of Vo'. *Nature.* **2020**. 584, 425-429 DOI: 10.1038/s41586-020-2488-1.
5. New Mexico Department of Health, COVID-19 in New Mexico. Available online: https://cvprovider.nmhealth.org/public-dashboard.html (accessed on 27 Aug 20201).
6. Li, Q.; Guan, X,; Wu, P.; Wang, X,; Zhou, L.; Tong, Y.; Ren, R.; Leung, K.S.M.; Lau, E.H.Y.; Wong, J.Y.; Xing, X.; Xiang, N.; Wu, Y.; Li, C.; Chen, Q.; Li, D.; Liu, T.; Zhao, J.; Liu, M.; Tu, W.; Chen, C.; Jin, L.; Yang, R.; Wang, Q.; Zhou, S.; Wang, R.; Liu, H.; Luo, Y.; Liu, Y.; Shao, G.; Li, H.; Tao, Z.; Yang, Y.; Deng, Z.; Liu, B.; Ma, Z.; Zhang, Y.; Shi, G.; Lam, T.T.Y.; Wu, J.T.; Gao, G.F.; Cowling, B.J.; Yang, B. Leung, G.M.; Feng, Z. Early Transmission Dynamics in Wuhan, China, of Novel Coronavirus-Infected Pneumonia. *N Engl J Med.* **2020,** *382:13*, 1199-1207. DOI: 10.1056/NEJMoa2001316.
7. Arons, M.M.; Hatfield, K.M.; Reddy, S.C.; Kimball, James, A.; Jacobs, J.R.; Taylor, J.; Spicer, K.; Bardossy, A.C.; Oakley, L.P.; Tanwar, S.; Dyal, J.W.; Harney, J.; Chisty, Z.; Bell, J.M.; Methner, M.; Paul, P.; Carlson, C.M.; McLaughlin, H.P.; Thornburg, N.; Tong, S.; Tamin, A.; Tao, Y.; Uehara, A.; Harcourt, J.; Clark, S.; Brostrom-Smith, C.; Page, L.C.; Kay, M.; Lewis, J.; Montgomery, P.; Stone, N.D.; Clark, T.A.; Honein, M.A.;

Duchin, , J.S.; Jernigan; J.S. for the Public Health-Seattle, K. County, and C. C.-. I. Team. Presymptomatic Sars-CoV-2 Infections and Transmission in a Skilled Nursing Facility. *NEJM*. **2020,** *382*, 2081-2090. DOI: 10.1056/NEJMoa2008457

8. Bullard, J.; Dust, K.; Funk, D.; Strong, J.E.; Alexander, D.; Garnett, L.; Boodman, C.; Bello, A.; Hedley, A.; Schffman, Z.; Doan, K.; Bastien, N.; Li, Y.; Van Caeseele, P.G.; Poliquin, G.. Predicting Infectious Severe Acute Respiratory Syndrome Coronavirus 2 from Diagnostic Samples. *Clinical Infectious Diseases*, **2020**, *71:*10, 2663-2666. DOI: DOI:10.1093/cid/ciaa638.
9. van Kampen, J.J.A.; van de Vijver, D.A.M.C.;Fraaij, P.L.A.; Haagmans, B.L.; Lamers, M.M.; Okba, N.; van den Akker, J.P.C.; Endeman, H.; Gommers, D.A.M.P.J.; Cornelissen, J.J.; Hoek, R.A.S.; van der Eerden, M.M.; Hesselink, D.A.; Metselaar, H.J.; Verbon, A.; de Steenwinkel, J.E.M.; Aron, G.I.; van Gorp, E.C.M.; van Boheemen, S.; Voermans, J.C.; Boucher,C.A.B.; Molenkamp, R.; Koopmans, M.P.G.; Geurtsvankessel, C.; van der Eijk, A.A. Shedding of Infectious Virus in Hospitalized Patients with Coronavirus Disease-2019 (COVID- 19). *Nat Commun* **2021**, *12*. DOI: 10.1038/s41467-020-20568-4
10. COVID-19 Prevalence Calculator. Available online: https://preventepidemics.org/covid19/resources/prevalence-calculator/#how-to-use-the-calculator (accessed on 03 June 2020).
11. Skrip, L.A.; Selvaraj, P.; Hagedorn, B.; Ouédraogo, A.L.; Noori, N.; Orcutt, A.; Mistry, D.; Bedson, J.; Hébert-Dufresne, L.; Scarpino, S.V.; Althouse, B.M. Seeding COVID-19 across Sub-Saharan Africa: An Analysis of Reported Importation Events across 49 Countries. *Am J Trop Med Hyg*. **2021,** 8, *104:5*, 1694–1702. DOI: 10.4269/ajtmh.20-1502.
12. Republic of Sierra Leone, Ministry of Health website. Available online: https://mohs.gov.sl/covid-19/. (accessed on 22 September 2020).
13. Adiga, A.; Venkatramanan, S.; Schlitt, J.; Peddireddy, A.; Dickerman, A.; Bura, A.; Warren, A.; Klahn, B.D.; Mao, C.; Xie, D.; Machi, D.; Raymond, E.; Meng, F.; Barrow, G.; Mortveit, H.; Chen, J.; Walke, J.; Goldstein, J.; Wilson, ML.; Orr, M.; Porebski, P.; Telionis, P.A.; Beckman, R.; Hoops, S.; Eubank, S.; Baek, Y.Y.; Lewis, B.; Marathe, M.; Barrett, C. Evaluating the Impact of International Airline Suspensions on the Early Global Spread of COVID-19. *medRxiv* Preprint. **2020**. *23:2020.02.20.20025882*. DOI: 10.1101/2020.20.20025882.
14. Brockmann, D.; Helbing, D. The hidden geometry of complex, network-driven contagion phenomena. *Science* **2013** *342.6164*, 1337-1342. DOI: 10.1126/science.1245200.
15. The World Factbook. Available online: https://www.cia.gov/the-world-factbook/ (accessed on 10 July 2020).
16. Hospital beds (per 1,000 people. Available online: https://data.worldbank.org/indicator/SH.MED.BEDS.ZS (accessed on 10 July 2020).
17. Dixon, M.G.; Taylor, M.M.; Dee, J.; Hakim, A.; Cantey, P.; Lim, T.; Bah, H.; Camara, S.M.; Ndongmo, C.B.; Togba, M.; Touré, L.Y.; Bilivogui, P.; Sylla, M.; Kinzer, M.; Coronado, F.; Tongren, J.E.; Swaminathan, M.; Mandigny, L.; Diallo, B.; Seyler, T.; Rondy, M.; Rodier, G.; Perea, W.A.; Dahl, B. Contact Tracing Activities during the Ebola Virus Disease Epidemic in Kindia and Faranah, Guinea, 2014. *Emerg Infect Dis*. **2015,** *21:11,* 2022-8. DOI:10.3201//eid2111.150684.
18. Olu O.O.; Lamunu, M.; Nanyunja, M.; Dafae, F.; Samba, T.; Sempiira, N.; Kuti-George, F.; Abebe, F.Z.; Sensasi, B.; Chimbaru, A.; Ganda, L.; Gausi, K.; Gilroy, S.; Mugume, J. Contact Tracing during an Outbreak of Ebola Virus Disease in the Western Area Districts of Sierra Leone: Lessons for Future Ebola Outbreak Response. *Front Public Health*. **2016**, *4:130*. DOI: 10.3389/fpubh.2016.00130.
19. Sierra Leone Ebola Lessons Shaping Massachusetts COVID-19 Response. Available online: https://www.pih.org/article/sierra-leone-ebola-lessons-shaping-massachusetts-covid-19-response (accessed on 15 July 2020).
20. Moore, M.; Gelfeld, B.; Okunogbe, A.T.; Paul, C. *Identifying Future Disease Hot Spots: Infectious Disease Vulnerability Index*. RAND Corporation: Santa Monica, CA, USA, 2016. Available online: https://www.rand.org/pubs/research_reports/RR1605.html (accessed on 01 June 2020).
21. Coronavirus (COVID-19) Vaccinations - Statistics and Research. Available online: https://ourworldindata.org/covid-vaccinations (accessed 14 June 2021).
